# Rates of coverage and determinants of complete vaccination of children in rural areas of Burkina Faso (1998-2003)

**DOI:** 10.1186/1471-2458-9-416

**Published:** 2009-11-17

**Authors:** Drissa Sia, Pierre Fournier, Jean-François Kobiané, Blaise K Sondo

**Affiliations:** 1 Département de médecine sociale et préventive, Université de Montréal, Québec, Canada; 2 Centre de recherche du CHUM, Université de Montréal, Québec, Canada; 3 Institut supérieur des sciences de la population, Université de Ouagadougou, Burkina Faso; 4 Institut de recherche en sciences de la santé, Ouagadougou, Burkina Faso

## Abstract

**Background:**

Burkina Faso's immunization program has benefited regularly from national and international support. However, national immunization coverage has been irregular, decreasing from 34.7% in 1993 to 29.3% in 1998, and then increasing to 43.9% in 2003. Undoubtedly, a variety of factors contributed to this pattern. This study aims to identify both individual and systemic factors associated with complete vaccination in 1998 and 2003 and relate them to variations in national and international policies and strategies on vaccination of rural Burkinabé children aged 12-23 months.

**Methods:**

Data from the 1998 and 2003 Demographic and Health Surveys and the Ministry of Health's 1997 and 2002 Statistical Yearbooks, as well as individual interviews with central and regional decision-makers and with field workers in Burkina's healthcare system, were used to carry out a multilevel study that included 805 children in 1998 and 1,360 children in 2003, aged 12-23 months, spread over 44 and 48 rural health districts respectively.

**Results:**

In rural areas, complete vaccination coverage went from 25.9% in 1998 to 41.2% in 2003. District resources had no significant effect on coverage and the impact of education declined over time. The factors that continued to have the greatest impact on coverage rates were poverty, with its various dimensions, and the utilization of other healthcare services. However, these factors do not explain the persistent differences in complete vaccination between districts. In 2003, despite a trend toward district homogenization, differences between health districts still accounted for a 7.4% variance in complete vaccination.

**Conclusion:**

Complete vaccination coverage of children is improving in a context of worsening poverty. Education no longer represents an advantage in relation to vaccination. Continuity from prenatal care to institutional delivery creates a loyalty to healthcare services and is the most significant and stable explanatory factor associated with complete vaccination of children. Healthcare service utilization is the result of a dynamic process of interaction between communities and the healthcare system; understanding this process is the key to understanding better the factors underlying the complete vaccination of children.

## Background

Vaccination is recognized as one of the most effective and efficient public health interventions. Vaccination campaigns carried out worldwide after the launch in 1974 of the Expanded Program on Immunization (EPI) contributed to the eradication of smallpox in 1979 and made possible the elimination of poliomyelitis on several continents and of neonatal tetanus in two-thirds of developing countries [1]. Remarkable progress was observed in the rate of vaccination coverage worldwide, going from 5% of the world's children having access to vaccination in 1974 to a steady level in 1990 of more than 70% average vaccination coverage worldwide in DTP3 (diphtheria, tetanus, pertussis, 3^rd ^dose). In sub-Saharan Africa this rate, which had reached 55% in the 1990s, declined to 53% in 2000. This trend was also noted in South Asia, thus exposing the disparities [2,3] concealed by the global average and the risks faced by millions of children in developing countries in relation to vaccination-preventable diseases [1].

Many initiatives aimed at increasing vaccination coverage particularly in low-income countries--the most recent (2000) of which is the Global Alliance for Vaccine and Immunization (GAVI)--have appeared at intervals of about five years [4], highlighting the difficulty of increasing and maintaining high levels of vaccination. Thus, a recovery of DTP3 coverage in the WHO African region was noted, reaching 69% in 2004--still under the 80% target. According to the authors, an increase in donor funding played a role in achieving this improvement in vaccination coverage [5-7]. Achieving and sustaining complete vaccination coverage of children are more crucial in rural than urban areas [1,8-11].

In Burkina Faso, a West African country that ranked 176 out of 177 in the 2007/2008 Human Development Index [12], complete vaccination coverage remains low and its progress irregular, with a regression from 34.7% in 1993 to 29.3% in 1998, and a subsequent rise to 43.9% in 2003 [8-10].

Burkina Faso's healthcare system has been marked by three periods corresponding to three different types of organization [13-15]:

- *From colonial times to the 1980s*, health policy was based on the fight against the major endemic diseases (smallpox, leprosy, onchocerciasis, trypanosomiasis, and potentially epidemic diseases). The healthcare system was organized into 10 medical sectors and immunization was ensured by a mobile program [16].

- *The period from 1980 to 1992 *was marked by a new policy based on primary healthcare, with a focus on bringing the supply of services closer to the rural population.

- *The period 1993 to today *is characterized by the implementation of health districts (53) based on the principle of management autonomy with cost recovery. The public health system has a pyramidal structure with three levels: central, regional and the health districts. The health district consists of two levels, the first of which is made up of the health centre (centre de santé et de promotion sociale - CSPS) (1051) and the second of medical centres with surgical units (CMA).

The CSPS, among its activities, provides vaccination to children and pregnant women. It has a dispensary and a maternity unit and is administered by a head nurse, assisted by a birth attendant (sometimes a midwife, either female or male), a mobile health officer and a matron.

The EPI has benefited from many types of support steadily since 1996 (see Table 3). Within the context of the Initiative for Vaccination Autonomy (IIV), Burkina Faso, following the example of other sub-Saharan countries, added a vaccination line item to its budget and signed, in June 1996, a cooperation agreement to have UNICEF supply vaccines and EPI materials [17]. To monitor the budgets allocated to the purchase of vaccines and consumables, the ARIVA project (*Appui au renforcement de l'indépendance vaccinale en Afrique*/Support for the strengthening of vaccination autonomy in Africa) was implemented in 1997 [18,19]. To deal with successive outbreaks of measles epidemics with high case fatality rates (4% to 8%) in 1996, 1998 and 1999, Burkina developed and implemented, in 1998, a plan for accelerated measles control [20].

In accordance with the policy of health system decentralization, the health districts have received, since 1999, funds from the State for their activities, among them vaccination [21,22].

To improve the safety of injections, self-blocking syringes and safety boxes were introduced in 1999 and subsequently applied to routine vaccination in 2002 [23,24].

In 2001, a National Health Development Plan was developed following round table talks among the funding agencies [22]. At the same time, a plan for social communication and mobilization was developed to complement the five-year EPI 2001-2005 plan, in recognition of the important role of communication in the different vaccination strategies that encompass routine vaccination, vaccination campaigns and the monitoring of EPI targeted and potentially epidemic diseases [20].

Despite these various reforms and the increased allocation of public funds to the health sector, utilization of curative health services, vaccination coverage and patient satisfaction with the public system have all regressed [13,25]. Researchers looking into the reasons for the poor performance of the reforms noted, as factors that might explain the situation, the content of the reforms, their implementation modalities, the context in which they are carried out and the actors involved or who have influence [15].

As individual factors associated with vaccination are extensively documented, the aim of this study is to identify both individual and systemic factors associated with complete vaccination in 1998 and 2003 and to relate them to variations in national and international policies and strategies on child vaccination. (For more details, see Additional file 1).

## Methods

### Study population

Our study population was children aged 12-23 months at the time of the survey [26]. In all, the study involved 805 children (898 after weighting) distributed among 44 rural health districts, for the year 1998, and 1,360 children (1,461 after weighting) distributed among 48 rural health districts, for the year 2003. During the DHS data analysis, to ensure representativeness of the sample, a weighting was applied that took into account the method of sampling [9,10]. An administrative reform increased the number of districts in 2003.

### Sources of data

Three data sources were used.

1. The Demographic and Health Surveys (DHS) of 1998-1999 and 2003 [9,10]: the sample, drawn from a random sampling stratified to two levels, is representative at both the national level and the level of residential areas (urban/rural). Burkina is divided into communes, which are basic territorial communities organized into sectors or villages. There are two types of communes, urban and rural. The rural commune is a collection of villages that has a combined population of at least 5,000 inhabitants and whose economic activities generate budgetary resources of at least 5 million CFA francs [27]. The data were collected through direct interviews in people's homes, and those related to vaccination were gathered from vaccination cards or mothers' statements in the absence of written documentation.

2. The Statistical Yearbooks of 1997 and 2002 [28,29]: These are prepared annually from reports and information gathered from all the health facilities and administrative structures in the country's healthcare system. They provide information on the country's health facilities coverage (structures and staffing) and health services utilization.

3. To better understand the evolution of the factors that might explain complete vaccination of children, we carried out individual interviews with decision-makers at the central and regional levels, as well as with field workers in Burkina's healthcare system. These were semi-directed, face-to-face interviews, recorded by the first author (DS) and transcribed under his supervision. We interviewed nine decision-makers at the central level and four at the regional level, three field workers, and five representatives of non-governmental organizations.

Ethical approval was granted by the *Comité d'éthique pour la recherche en santé of Burkina Faso *(agreement N° 2007-056 from November 8, 2007).

### Variables

The dependent variable is the child's vaccination status, which is dichotomous: the child is either completely vaccinated or not. Completely vaccinated children are those who have received the BCG, measles, and yellow fever vaccines as well as the three doses of DTP and oral polio vaccines during their first year of life, according to either their vaccination cards or their mothers' statement.

For the explanatory variables, given the hierarchical structure of the data, two levels were taken into consideration:

- Level 1 variables (characteristics of the child and family environment) including: personal characteristics (mother's religion, parents' professional group and level of education, mother's use of at least one source of information, birth rank, sex of the child); experience with the utilization of maternal-child services (continuity from prenatal care to institutional delivery); household characteristics (number of mother's co-wives, number of other children aged five and under, number of children deceased, and standard of living, for which we used a proxy based on household goods and characteristics of the dwelling [26]). The 2003 DHS included new variables, and two of them--the mother's childhood place of residence (urban or rural) and whether she had the possibility of deciding to use medical services if ever the child was ill--were taken into consideration.

- Level 2 variables (health district): percentage of educated women, population/vaccinator ratio, average catchment area of the CSPSs and population/CSPS ratio.

### Statistical analyses

We applied the same approach used in the analysis of the 1998 data [26] to those of 2003:

- an analysis between each explanatory variable and the dependent variable to sort the variables;

- a level-1 logistical regression to determine the odds ratios and their 95% confidence intervals for the variables at this level;

- a multilevel analysis to construct the model, consisting of a logit-type statistical formalization, before adjusting the model (in five steps); for this, the estimation of parameters began with the first order of marginal quasi-likelihood (MQL), then the estimates obtained were improved with the second order of predictive quasi-likelihood (PQL). The restrictive iterative generalized least squares (RIGLS) method was used because of the small size of the level-2 units (44 districts in 1998 and 48 in 2003).

## Results

### Comparison of factors associated with complete vaccination of children, determined by logistical regression in 1998 and 2003

Table 1 presents the variables selected to model the complete vaccination of children (univariate analyses).

**Table 1:** Complete vaccination of children aged 12-23 months and variables retained in 1998 (n = 898) and 2003 (n = 1461) (univariate analyses)

Variables	Children aged 12-23 months with complete vaccinations			
	
	1998		2003	
	
	Proportion (%)*	Adjusted OR** (CI 95%)	Proportion (%)*	Adjusted OR** (CI 95%)
**Mother able to decide to use a medical treatment when child is ill**^*a*^				
No, or it depends (n_03 _= 402)	-	-	27.1	1
Yes (n_03 _= 1059)	-	-	39.8	1.27 (0.97 - 1.67)
**Mother's education**				
No schooling (n_98 _= 850)	20.2	1	-	-
Primary or secondary school (n_98 _= 48)	37.5	1.52 (0.74-3.11)	-	-
**Education of the mother's partner**				
No schooling (n_98 _= 829; n_03 _= 1321)	19.8	1	34.8	1
Primary or secondary school and more (n_98 _= 46; n_03 _= 107)	39.1	1.41 (0.69-2.88)	51.4	1.27 (0.83 - 1.93)
No information supplied (n_98 _= 23; n_03 _= 34)	34.8		50.0	
**Utilization of sources of information (radio, television, newspaper)**				
No source (n_98 _= 756)	19.4	1	-	-
At least one of the sources (n_98 _= 142)	30.3	1.26 (0.78-2.04)	-	-
**Mother's occupation**				
Does not work (n_98 _= 162)	20.4	1	-	-
Agriculture (n_98 _= 403)	17.1	0.81 (0.48-1.37)	-	-
Other employment (n_98 _= 332)	26.5	1.28 (0.77-2.14)	-	-
**Occupation of the mother's partner**				
Other (n_98 _= 107)	30.8	1.12 (0.65-1.91)	-	-
Agriculture (n_98_791)	19.8	1	-	-
**Mother's religion**				
Other (n_03 _= 182)	-	-	26.4	1
Muslim (n_03 _= 942)	-	-	35.1	1.14 (0.80 - 1.63)
Christian (n_03 _= 338)	-	-	45.0	1.34 (0.90 - 1.99)
**Number of mother's co-wives**				
No co-wives (n_03 _= 753)	-	-	39.0	
One co-wife (n_03 _= 442)	-	-	35.7	
Two co-wives (n_03 _= 266)	-	-	29.7	
**Number of other children aged 5 and under**				
Foor children or more (n_03 _= 363)	-	-	28.4	1
Three children (n_03 _= 252)	-	-	35.3	1.33 (0.92 - 1.92)
Two children (n_03 _= 526)	-	-	38.2	1.39 (1.01 - 1.91)
One child at most (n_03 _= 320)	-	-	43.1	1.75 (1.23 - 2.49)
**Standard of living**				
Poor (n_98 _= 298; n_03 _= 368)	14.8	1	30.2	1
Well-off (n_98 _= 599; n_03 _= 1092)	24.4	1.69 (1.11-2.57)	38.4	1.27 (0.96 - 1.69)
**Continuity from prenatal care to assisted delivery**				
Neither PNC or assisted delivery (n_98 _= 341; n_03 _= 365)	8.5	1	14.4	1
PNC or assisted delivery (n_98 _= 306; n_03 _= 659)	22.2	2.91 (1.76-4.81)	38.8	3.23 (2.26 - 4.62)
PNC and assisted delivery (n_98 _= 252; n_03 _= 437)	37.3	5.11 (3.06-8.52)	50.8	5.65 (3.86 - 8.26)

* All proportions are significant to 5%.

** Logistic regression with level 1 significant variables.

N_98 _= number of persons in 1998; n_03 _= number of persons in 2003.

^*a*^Not available in the 1998 database.

In 1998 a child from a well-off household was 1.7 times more likely to be completely vaccinated compared to one from a poor household, but in 2003 the standard of living did not appear to have an effect on the probability of being completely vaccinated. In 2003, having fewer children in the household was associated with a higher probability of being vaccinated. This factor was not associated with complete vaccination in 1998. In addition, the positive relationship observed in 1998 between complete vaccination of children and continuity from prenatal care to institutional delivery appeared even more marked in 2003.

### Systematic variation of complete vaccination in 1998 and in 2003

Variance analysis of a factor with random effects showed systematic variation in full vaccination both in 1998 (χ^2 ^= 11.262 to one degree of freedom (DF) with p = 0.00079) and in 2003 (χ^2 ^= 9.167 to one DF with p = 0.0024642). However, the proportion of this variation that is attributable to differences between health districts is five times less in 2003 (7.5%) than was seen in 1998 (37.14%).

### Comparison of factors associated with complete vaccination of children, determined by multilevel analysis in 1998 and 2003 (final model)

Table 2 presents the variables associated with complete vaccination of children (multilevel analysis).

**Table 2:** Factors associated with complete vaccination of children aged 12-23 months in 1998 and 2003 (multivariable analyses)

Variables	Child completely vaccinated			
	
	1998		2003	
	
	Model 1	Model 2	Model 1	Model 2
	
	OR (CI 95%)	OR (CI 95%)	OR (CI 95%)	OR (CI 95%)
**Standard of living**				
Poor	1	1	-	-
Well-off	1.9 (1.17-3.08)	1.88 (1.15-3.06)	- -	- -
**Number of other children aged 5 or under**				
Four children or more	-	-	1	1
Three children	-	-	1.20 (0.84 - 1.73)	1.19 (0.83 - 1.72)
Two children	-	-	1.36 (1.01 - 1.86)	1.35 (1.01 - 1.82)
One child at most	-	-	1.77 (1.26 - 2.48)	1.76 (1.25 - 2.47)
**Continuity from prenatal care to assisted delivery**				
Neither PNC nor institutional delivery	1	1	1	1
PNC or institutional delivery	3.13 (1.77-5.55)	3.04 (1.71-5.4)	3.44 (2.44 - 4.85)	3.42 (2.43 - 4.82)
PNC and institutional delivery	5.98 (3.36-10.64)	5.64 (3.16-10.05)	6.34 (4.42 - 9.09)	6.18 (4.30 - 8.89)
**District level variable**				
Proportion of educated women by district	-	1.14 (1.01-1.27)	-	1.03 (0.99 - 1.08)

In 1998, residual variances of complete vaccination were 1.94, 1.673, and 1.543, respectively, for model 0 (without predictor, with coefficient of -1.829), model 1 and model 2.

In 2003, residual variances of complete vaccination were 0.267, 0.226, and 0.217, respectively, for the constant-only model, model 1 and model 2.

In both 1998 and 2003 the effects of the level 1 variables did not vary by health district and the models constructed are random intercept models.

At level 1, continuity from prenatal care to assisted delivery remains associated with vaccination status in both 1998 and 2003. In 1998 the standard of living was associated with vaccination status while in 2003 the number of other children aged five or under became significantly linked with complete vaccination.

At level 2, in 1998 each one-unit (i.e., 1%) increase in the proportion of educated women in a health district increased children's chances of being completely vaccinated by a factor of 1.14; in 2003 the impact of the proportion of educated women in a health district was not significant. In both 1998 and 2003, no interaction was found among the variables of level 1, nor between variables of levels 1 and 2 (Table 2). Variables related to district resources (mean number of inhabitants per vaccinator staff) and to accessibility (average catchment area, mean number of inhabitants per CSPS) had no significant impact on complete vaccination in either 1998 or 2003.

### Comparison of complete vaccination coverage of children aged 12-23 months in 1998 and 2003 and key events for EPI

Even if complete vaccination coverage has not yet reached the desired level within this context of multiple vaccination-promotion initiatives, it did increase significantly in 2003 (41.2%) in comparison with 1998 (25.9%), with a chi-square of 60.39 to one degree of freedom and p < 0.000001.

Table 3 presents the key initiatives undertaken to promote vaccination.

**Table 3:** Factors and trends associated with complete vaccination coverage in rural areas, in the context of multiple initiatives

Key events	Complete vaccination coverage	Factors associated with complete coverage
**1993**	**29%**	
	
1994 - Creation of health districts		
		
1994 - Devaluation of CFA franc		
		
1996 - Decentralization of public healthcare system		- Standard of living
		
1996 - National Vaccination Day 1		- Continuity of prenatal care and assisted delivery
		
1996 - Cooperation agreement with UNICEF to supply vaccines and consumables		- % of educated women
		
1996 - National Vaccination Day 2		
		
1997 - ARIVA project		
		
1998 - Implementation of measles control plan		

**1998/1999 -**	**26%**	
	
1999 - Funds allocated to health districts by the State		
		
1999 - National Vaccination Day 2 and introduction of self-blocking syringes and safety boxes		- Continuity of prenatal care and assisted delivery
		
2000 - Debt reduction through the HIPC initiative		- Number of other children aged 5 or under
		
2000 - Cost of syringes assumed by SPV and cost of vaccination cards by CoGes		
		
2000 - GAVI funding available		
		
2000 - Revival of the CCIA		
		
2001 - Adoption of the National Health Development Plan (PNDS)		
		
2001 - Strategic plan for social mobilization		
		
2001 - GAVI funding to strengthen the health system		
	
**2003**	**41%**	

## Discussion

This study shows that complete vaccination coverage of children increased significantly between 1998 and 2003 and disparities in coverage between districts diminished. The factors that continued to have the greatest impact on these coverage rates were poverty, with its various dimensions, and the utilization of healthcare services.

### Increase in vaccination coverage

The significant increase in complete vaccination coverage seen in 2003 raises certain points for discussion:

- The addition of financial resources into the healthcare system has the effect of increasing vaccination coverage when it is low [5]. In fact, the authors showed that GAVI support helps to increase DTP3 coverage in countries where the rate of coverage is below 65% [30]. As can be seen in Table 3, between 1997 and 2001 new initiatives to support vaccination were implemented every year. It seems logical to consider the cumulative impact of these interventions, coming one after the other and leading to a significant increase in vaccination coverage. In fact, the positive impact on children of vaccination initiatives is widely recognized. The major difficulty is in sustaining them, as is illustrated by the statement of a person who had worked at all levels in the system (peripheral, regional and central): "*The challenge continues to be in maintaining the level of vaccination coverage, because these initiatives come along, create an increase in vaccination coverage, and when they are gone, coverage falls back again*."

- The decentralization of the healthcare system and of EPI management, which introduced changes in practice, could have improved both the technical and perceived quality of healthcare services [31]. In fact, an analysis of healthcare services utilization from 1986 to 1997 had indicated the need for in-depth reform of Burkina's healthcare system, particularly in the areas of human resources, funding policy and management (sectoral approach instead of the project approach, communication). The authors saw decentralization as an opportunity to improve the healthcare system [13]. Because the focus for some time was on the sectoral approach, with the revival of the Inter-agency Coordinating Committee and the implementation of decentralization by creating communes and involving the population in the management of health centres via management committees, we would expect to see an increase in the use of services, including vaccination services [see Table 3]. In fact, after an overall decrease in the rate of healthcare services utilization observed between 1984 and 1998 that led the authors [14] to conclude the reforms had failed, an increasing trend was observed from 1998 on, suggesting that the increase in the rate of child vaccination over the same period is not an isolated case.

- Measures were taken to ensure the safety of injections, thus reducing post-immunization reactions. One decision-maker at the central level observed: "*The staff is well trained and qualified, and Burkina is one of the few countries where the cold chain meets WHO standards*."

- There were nevertheless reservations expressed regarding improving the quality of services, particularly the perceived quality [32]. Assurances about the quality of training provided to vaccination workers are nuanced at the regional level. In fact, the districts plan training sessions for their new workers (coming out of school) to prepare them to carry out vaccination strategies. This is illustrated by the statements of a regional administrator in response to a question about the late arrival of various funds allocated to the health districts: "*Yes, for example, this year there are districts that had planned training sessions for their new vaccinators. So, these workers will not be trained. They came out of school because this is not often offered in the school's courses. So they won't be trained*."

### Reduction in vaccination coverage disparities

Differences between health districts in relation to complete vaccination of children tended to lessen between 1998 and 2003. In fact, even though the healthcare system was decentralized after the creation of health districts in 1994 [33], with district management teams being given a certain amount of autonomy, the central authority at the Ministry of Health retained a major role in decision-making. Among the domains kept at that level were the supply of vaccines and consumables, funding, equipment for the cold chain and rolling stock, and, to a lesser extent, the regulation and distribution of development partners whenever possible, as was revealed by the various actors in the individual interviews. It must be noted that vaccination is funded within a normative framework, based on action plans developed by the health districts. The functioning of this regulatory framework for funding vaccination activities in health districts is recognized and accepted by all actors in the healthcare system (central, regional and district levels, NGOs). This is illustrated by the statements of various resource persons. One NGO representative explained: "*At the beginning of the year, we inform the health districts and the regional health administrations which activities will be funded. This way, people know what activities to put into their action plans to get them funded*." A manager at the district level spoke along the same lines: "*Our action plan is based on a planning framework (directives) from the central authority. These directives tell us that this year, for vaccination, here's how much you can put in as activities that are in accordance with national policy. Then it is adopted by the board of the health district*." He added that, "*the funding agencies, through a concerted action framework, are already in agreement with the central authority, and when they come to the resource allocation session, there isn't much discussion*."

This involvement of the central government has the benefit of making the districts comparable in terms of funding and supplies of material resources and, to a lesser extent, in terms of organization. On the other hand, decisions taken at the central government level, by means of the planning framework sent to the health districts and the specifications imposed by development partners regarding activities they are prepared to fund, leave little room for each district to resolve specific local problems, which will not be included in the health districts' action plans because they are not targeted by funding agencies. Over time, this situation can discourage actors at the operational level from developing any initiatives. For example, if there is a delay in receiving funding, actors in the field have no alternative but to adjust their action plans, even if they would be able to pre-finance the activity concerned, as is recounted by one actor at the regional level: "*The Ministry of Health no longer allows pre-financing, which means the districts have to revise their action plans. This affects the morale of the workers and also, indirectly, vaccination, and especially its quality*." Another case in point is the fight against meningitis; a person at the central level told: "*It was recommended that the regions and health districts should be able to include, in their action plans, a certain number of activities related to fighting meningitis epidemics. These epidemics had become so frequent that they were listed as priority issues in all the districts. Unfortunately, this still hasn't been done, but we continue to ask for these activities to be included in the health districts' action plans so that they can benefit from some funding sessions." *In fact, the resolution of problems that are specific to each district in general, and to the area of each CSPS in particular, is the responsibility of the health centres' management committees; as was said by a regional administrator, "*the operational funding of vaccination is local*." It seems reasonable to assume that the remaining differences between health districts could be explained, in part, by the community's involvement in these districts' health centres and by whether or not the management committee, which in principle reflects the community, functions well.

### Poverty

Both in 1998 and in 2003, our results confirm that poverty and resource constraints are an impediment to complete vaccination of children in rural areas [34]. Independently of poverty, in 2003, we found a link between the number of children aged five and under in the household and the probability of being vaccinated. It may be that in five years the situation has become more worrisome [35], such that despite vaccination being free [20], households have become more careful about the use of family resources, which affects the priority given to children and increasingly takes into account the opportunity costs related to wait times, missed appointments, and absence from work in the fields and in the market. Continued poverty would motivate households to ration resources more carefully as the number of children grows [36].

### Experience of healthcare services utilization

The experience of healthcare (maternity) services utilization by the mother appears to be the most stable and strongest explanatory factor for complete vaccination of children. Mothers' loyalty to vaccination services depends on the quality of that experience. In fact, it was this concept of loyalty that led to using the DTP3 rate to assess the performance of vaccination services and thus their capacity to retain their users [37].

### Education

In this study, factors related to education did not appear to play a fundamental role in complete vaccination of children. In 1998, the community's level of education appeared to be an explanatory factor for complete vaccination in children, but this was no longer the case in 2003. Our observation differs from what has been reported in other contexts [38-42]. This reflects an increasing popularization of vaccination in a population that is becoming more organized into associations that deal with specific issues and, at the same time, become useful channels of communication. In addition, vaccination's benefits and positive effects are widely recognized. People attribute the drop in cases of measles and poliomyelitis to vaccination, and when they speak, for example, of poliomyelitis, they refer to cases of paralysis in the past, noting that these have become rare thanks to the drops that children receive. The following statements from resource persons illustrate this. According to a decision maker at the central level, "*Before, when you entered many homes, you saw poliomyelitic children dragging along, and this was frequent. But the disabled persons we encounter today are of a certain age, they are no longer the very youngest ones; that has become the exception. Measles was very common. In our region, there was an adage that if your child had not yet had measles, he was not yours. It was even said that, if you hadn't had measles by the time you died, you would get it in your tomb--all to say, measles was inevitable. But we have seen that with vaccination, it is avoidable, and its impact in terms of morbidity and mortality has greatly diminished. All these observations have been made in recent years*." An administrator at the regional level said, "*I had both pertussis and measles, because I wasn't vaccinated. But today our children don't know what pertussis is, and measles has become sporadic*." In such a context, it seems reasonable that a mother's ability to decide for herself to use curative medical services creates a favourable situation for vaccination, which, moreover, has been declared a free service [20].

## Conclusion

Between 1998 and 2003, the rate of complete vaccination of children aged 12-23 months increased significantly, going from 25.9% to 41.2%. An analysis of the factors that could explain complete vaccination of children in these two periods reveals the following:

- Differences between districts are becoming fewer, making districts more comparable over time.

- Communities continue to live in poverty, and having more children in household results in the adoption of survival strategies, including rationing of available resources.

- Education, both at the individual and community levels, is no longer associated with complete vaccination of children.

- Continuity from prenatal care to institutional delivery creates a loyalty to these services among the population and is the most significant and most stable explanatory factor associated with complete vaccination of children.

The utilization of healthcare services in general, and of prenatal services in particular, is the result of a relationship between the community and the healthcare system. Vaccination coverage is the result of a dynamic process that includes the experience of interaction between people and vaccination services, the belief or disbelief in the power of vaccination to protect against diseases, and its acceptance by the populations. Our study confirms the conclusions reached by other authors about the mechanisms underlying the evolution of vaccination coverage [43].

## Competing interests

The authors declare that they have no competing interests.

## Authors' contributions

DS participated in the study design, data collection, analysis, and interpretation of results. He drafted the first version of the article and participated in its revision. PF was involved in the study design, analysis and interpretation of results, and in writing the article. JFK was involved in the analysis and interpretation of results, and in revising the article. BKS was involved in the study design, data collection, and revising the article.

All authors read and approved the final manuscript.

## Pre-publication history

The pre-publication history for this paper can be accessed here:

http://www.biomedcentral.com/1471-2458/9/416/prepub

## Supplementary Material

Additional file 1 **Immunization program and health system in Burkina Faso: an historical overview**. Than the background section in the article, this document gives more details about historical evolution of the health system and immunization programs. Click here for file
