# Supplementary material for: Rates of coverage and determinants of complete vaccination of children in rural areas of Burkina Faso (1998-2003)
Source: BMC Public Health. 2009 Nov 17;9:416. doi: 10.1186/1471-2458-9-416 (PMC2784775; doi:10.1186/1471-2458-9-416)
Supplement: Additional file 1 — Immunization program and health system in Burkina Faso: an historical overview. Than the background section in the article, this document gives more details about historical evolution of the health system and immunization programs. [file 1471-2458-9-416-S1.DOC]

# Immunization program and health system in Burkina Faso: an historical overview

Vaccination is recognized as one of the most effective and efficient public health interventions. National vaccination campaigns carried out worldwide after the launch in 1974 of the Expanded Program on Immunization (EPI) contributed to the eradication of smallpox in 1979 and have made possible the elimination of poliomyelitis on several continents, and of neonatal tetanus in two-thirds of developing countries [1]. Remarkable progress was observed in the rate of vaccination coverage worldwide, going from 5% of the world’s children having access to vaccination in 1974, to a steady level in 1990 of more than 70% average vaccination coverage worldwide in DTP3 (diphtheria, tetanus, pertussis, 3^rd^ dose). During this same period, in sub-Saharan Africa, the rate of DTP3 vaccination coverage, which had reached 55% in the 1990s, declined to 53% in 2000. At the same time, this trend was also noted in South Asia, thus exposing the disparities [2, 3] concealed by the global average and the risks faced by millions of children in developing countries in relation to vaccination-preventable diseases [1].

In view of this situation, many initiatives aimed at increasing vaccination coverage, particularly in low-income countries—the most recent (2000) of which is the Global Alliance for Vaccine and Immunization (GAVI)—have appeared at intervals of about five years [4], highlighting the difficulty of increasing and maintaining high levels of vaccination. Thus, a recovery of DTP3 coverage in the WHO African region was noted as of 2000, reaching 69% in 2004—still under the 80% target. According to the authors, an increase in donor funding played a role in achieving this improvement in vaccination coverage [5-7].

Achieving and sustaining complete vaccination coverage of children are more crucial in rural than urban areas [1, 8-11], although the individual factors associated with this complete vaccination of children in rural areas are relatively well known. Among these factors are households’ standard of living and mothers’ utilization of prenatal services [12]. This latter factor requires that the healthcare system be accessible and credible to the population it serves. In Eastern European countries, where the dismantling of the Soviet Union entailed major upheavals of the healthcare system that damaged its credibility, a sharp drop in vaccination coverage was observed [1].

In Burkina Faso, a West African country that ranked 176 out of 177 in the 2007/2008 Human Development Index [13], complete vaccination coverage remains low and its progress irregular, with a regression from 34.7% in 1993 to 29.3% in 1998, and a subsequent rise to 43.9% in 2003 [8-10]. Are these variances attributable to factors related to population characteristics, or to changes in the healthcare system, or to a combination of both?

Burkina Faso’s healthcare system has been marked by three periods corresponding to three different types of organization [14-16]:

- *From colonial times to the 1980s*, health policy, based on the fight against the major endemic diseases (smallpox, leprosy, onchocerciasis, trypanosomiasis, and potentially epidemic diseases) was supported by health care provided free of charge by public health services provided by colonial troops, hospitals, and police health services. In this period, the healthcare system was organized into 10 medical sectors centred around the administrative departments, and immunization was ensured by a program of prospective vaccination [17].
- *The period from 1980 to 1992* was marked by a policy based on primary care, with a focus on bringing the offer of services closer to the rural population, while also reducing indirect and direct costs of access to services. Several initiatives were undertaken to strengthen primary care services: the “one village, one primary health post” campaign (1985), adherence to the Bamako Initiative (1992), and an attempt at collaboration between modern and traditional medicine. Hospital autonomy also emerged during this period. With 10 departmental administrations, the organization of the healthcare system was modelled on the country’s administrative organization.
- *The period 1993 to today* is characterized by the implementation of health districts (53 in number), in accordance with the recommendations of the Harare Conference (1987). The creation of districts was based on the principle of management autonomy with cost recovery. This principle was given concrete expression by the creation in each health and social services centre (CSPS) of an essential generic medicine (EGM) depot and the establishment of a management committee composed primarily of members of the community served by the CSPS. Other decisions were also introduced, such as the hospital reform that transformed hospitals into establishments under public administration (EPA), the reform of the pharmaceutical sector, and the liberalization of the health sector that resulted in an increase in the number of private facilities. The public health system, in a pyramidal structure, has three levels of responsibility: the central administration, under the authority of the Minister’s Cabinet, which is responsible for applying national policy; the intermediate level, under the regional health administrations, each headed by a regional director; and the peripheral level, under the authority of the health districts, which are headed by a district medical officer. The health district is thus the most decentralized operational entity of the national health system and consists of two levels, the first of which is made up of the CSPSs, and the second by medical centres with surgical units (CMA).

Burkina’s basic health service structure is the CSPS, of which there are 1,051 [18]. The CSPS, among its other activities, provides vaccination to children and pregnant women. The CSPS has a dispensary and a maternity unit and is administered by a head nurse for the post, assisted by a birth attendant (sometimes a midwife, either female or male), a mobile health officer and a matron.

The CMAs, of which there are 36, are managed by physicians trained in essential surgery, supported by surgical assistants and anaesthesia assistants [18]. They are the first line of referral providing, among other services, caesareans, hernia treatment and appendectomies.

Regional hospitals (nine in total), the second line of referral, receive medical evacuation cases from the districts in their region. The highest levels of referral are sent to the three national hospitals.

The healthcare system is funded by a combination of private funding from households, public funding from the state, and external aid. It is important to note that the 1994 devaluation of the CFA franc did not inhibit the growth in funding for the health sector [15].

The Expanded Program of Immunization (EPI) that began in the second period of the health system’s evolution is carried out in the CSPSs, and the structure charged with its management was promoted from a service to a department, being responsible for, among other things, coordinating activities and mobilizing external resources for the vaccination sub-sector [19]. The EPI has benefited from many types of support steadily since 1996. Within the context of the Initiative for Vaccination Autonomy (IIV), Burkina Faso, following the example of other sub-Saharan countries, added a vaccination line item to its budget and signed, in June 1996, a cooperation agreement to have UNICEF supply vaccines and EPI materials [20]. To monitor the allocation and execution of budgets consecrated to the purchase of vaccines and consumables in the national budgets, the ARIVA project (*Appui au renforcement de l’indépendance vaccinale en Afrique* / Support for the strengthening of vaccination autonomy in Africa) was implemented in 1997 [19, 21].

To deal with successive outbreaks of measles epidemics with high lethality (4% to 8%), in 1996, 1998 and 1999, Burkina developed and implemented, in 1998, a plan for accelerated measles control [22] based on three strategies:

- strengthening the routine EPI;
- continued supplementary mass vaccinations;
- strengthening the integrated monitoring of EPI targeted diseases such as measles.

In accordance with the policy of health system decentralization, the health districts have received, since 1999, funds from the State for their activities, among them vaccination [23, 24].

To improve the safety of injections, self-blocking syringes and safety boxes were introduced in the vaccination services during the second round of national vaccination days in 1999. This policy on injection safety was subsequently applied to routine vaccination in 2002 [25, 26].

The year 2000 saw many initiatives. In fact, within the context of the Heavily Indebted Poor Countries (HIPC) initiative, as the country reduced its debt, it directed these funds, as of 2000, first and foremost to funding the health sectors, in particular the EPI and education, as part of its poverty reduction strategy [24, 25]. Also in that year, the country was eligible for funding from GAVI to reinforce routine vaccination. These funds were available as of 2001 [27]. Vaccination was then declared a free service as of October 2000. To make this measure possible, the costs of consumables (syringes and needles) were assumed by the Service for Prevention by Vaccination (SPV), and that of the vaccination cards was covered by the management committees (CoGes) of the health posts [22]. Given the variety of partners and institutions (including NGOs and research institutes) supporting vaccination in the country, an Inter-agency Coordinating Committee (CCIA) that had been created in September 1989 was revived in July 2000. As an instrument for coordination, orientation, and resource mobilization, chaired by the Secretary General of the Ministry of Health, the CCIA has three main functions:

- ensure cooperation between partners working in the field of vaccination and the national party, to identify the best strategies for achieving the objectives;
- support the program’s implementation of planned activities by providing human and material resources, and mobilizing the required operating funds.
- adopt plans and strategic orientations for improving program management and achieving objectives [27].

In 2001, a National Health Development Plan was developed following round table talks among the funding agencies [24]. In that same year, a plan for social communication and mobilization was developed to complement the five-year EPI 2001-2005 plan, in recognition of the important role of communication in the different vaccination strategies that encompass routine vaccination, vaccination campaigns and the monitoring of EPI targeted and potentially epidemic diseases [22].

Burkina’s health system has thus undergone many reforms. Despite these various reforms and the increased allocation of public funds to the health sector, utilization of curative health services, vaccination coverage and patient satisfaction with the public system have all regressed [14, 28]. Meunier [28] showed that recourse to modern medicine did not become systematic for all individuals and that there are many issues at play, such as the type of illness, relationships established with the nurse, distance from the CSPS, financial accessibility, and the sick person’s age. Researchers looking into the reasons for the poor performance of the reforms noted, as factors that might explain the situation: the content of the reforms, the implementation modalities, the context in which they are carried out, and the actors involved or who have influence [16].

The aim of this study is to identify individual and environmental factors associated with complete vaccination in 1998 and 2003 and to relate their evolution to variations in national and international policies and strategies on child vaccination.

# References

1. World Health Organization: *State of the World's Vaccines and Immunization.* Geneva; 2003.

2. Barker LE, Chu SY, Li Q, Shaw KM, Santoli JM: **Disparities between white and African-American children in immunization coverage.** *J Natl Med Assoc* 2006, **98**:130-135.

3. Meheus F, Van Doorslaer E: **Achieving better measles immunization in developing countries: does higher coverage imply lower inequality?** *Soc Sci Med* 2008; **66**:1709-1718.

4. Hardon A, Blume S: **Shifts in global immunisation goals (1984-2004): unfinished agendas and mixed results.** *Soc Sci Med* 2005; **60**:345-356.

5. Arevshatian L, Clements CJ, Lwanga SK, Misore AO, Ndumbe P, Seward JF, Taylor P: **An evaluation of infant immunization in Africa: is a transformation in progress?** *B World Health Organ* 2007; **85**:449-457.

6. Johnson KA, Sardell A, Richards B: **Federal immunization policy and funding: a history of responding to crises.** *Am J Prev Med* 2000; **19**:99-112.

7. Szilagyi PG, Humiston SG, Shone LP, Barth R, Kolasa MS, Rodewald LE: **Impact of vaccine financing on vaccinations delivered by health department clinics.** *Am J Public Health* 2000; **90**:739-745.

8. Institut national de la statistique et de la démographie, Macro International Inc.: *Enquête démographique et de santé 1993, Burkina Faso*. Calverton, MD, USA: Macro International Inc.; 1994.

9. Institut national de la statistique et de la démographie, Macro International Inc.: *Enquête démographique et de santé, Burkina Faso 1998-99.* Calverton, MD, USA: Macro International Inc.; 2000.

10. Institut national de la statistique et de la démographie, ORC Macro: *Enquête démographique et de santé 2003, Burkina Faso*. Calverton, MD, USA: ORC Macro; 2004.

11. Noor AM, Amin AA, Gething PW, Atkinson PM, Hay SI, Snow RW: **Modelling distances travelled to government health services in Kenya.** *Trop Med Int Health* 2006; **11**:188-196.

12. Sia D, Kobiané JF, Sondo BK, Fournier P: **Les facteurs individuels et du milieu de vie associés à la vaccination complète des enfants en milieu rural au Burkina Faso: une approche multiniveau.** *Cahiers Santé* 2007; **17**:201-206.

13. United Nations Development Programme: *Human Development Report 2007/2008*. New York; 2007.

14. Bodart C, Servais G, Mohamed YL, Schmidt-Ehry B: **The influence of health sector reform and external assistance in Burkina Faso.** *Health Policy Plan* 2001; **16**:74-86.

15. Haddad S, Nougtara A, Fournier P: **Learning from health system reforms: lessons from Burkina Faso.** *Trop Med Int Health* 2006; **11**:1889-1897.

16. Nitiema AP, Ridde V, Girard J: **The efficiency of public health policies in West Africa: the case of Burkina-Faso.** *Int Polit Sci Rev* 2003; **24**:237-256.

17. Martin-Samos F: *Evolution de la situation médico-sanitaire en Haute Volta: essai d'analyse pour l'utilisation rationnelle des données de routine*. Upper Volta: Office of the WHO Program Coordinator for Upper Volta; 1982.

18. Direction des études et de la planification du Ministère de la Santé: *Annuaire statistique 2002*. Burkina Faso; 2002.

19. ARIVA: *Réunion Inter-Pays sur l'IIV à Bamako, du 7 au 9 mars 2005: Rapport de synthèse*. Burkina Faso; 2005.

20. Service de prévention par les vaccinations du Ministère de la Santé: *Programme Élargi de Vaccination (PEV): Recherche sur la participation communautaire et le financement des activités - rapport provisoire.* Burkina Faso; 2003.

21. ARIVA: **ARIVA**–**GAVI**–**UNICEF, le partenariat se renforce autour de la vaccination.** *ARIVA INFO* 2003-2004; **12:**1-19.

22. Ministère de la Santé: *Plan stratégique de communication sociale / mobilisation sociale pour le PEV au Burkina Faso 2001 - 2005*. Burkina Faso; 2001.

23. Ministère de la Santé: *Document de politique sanitaire nationale*. Burkina Faso; 2000.

24. Ministère des Finances et du Budget: *Table ronde des bailleurs de fonds du Plan National de Développement Sanitaire (PNDS) 2001 - 2010: Document de base*. Burkina Faso; 2003.

25. Service de la prévention par la vaccination du Ministère de la Santé: *Plan quinquennal national PEV Burkina Faso (2001*–*2005)*. Burkina Faso; 2000.

26. Service de la prévention par les vaccinations du Ministère de la Santé: *Déclaration de politique générale sur la sécurité des injections au Burkina Faso*. Burkina Faso; 2002.

27. Government of Burkina Faso: *Proposition de soutien soumise à l'Alliance mondiale pour les vaccins et la vaccination (GAVI) et aux fonds pour les vaccins*. Burkina Faso; 2003.

28. Meunier, A: *Système de soins au Burkina Faso: le paradoxe sanitaire*. Paris: L'Harmattan; 1999.
